# Enterogenic *Stenotrophomonas maltophilia* migrates to the mammary gland to induce mastitis by activating the calcium-ROS-AMPK-mTOR-autophagy pathway

**DOI:** 10.1186/s40104-023-00952-y

**Published:** 2023-12-20

**Authors:** Zhaoqi He, Caijun Zhao, Yuhong He, Zhuoyu Liu, Guyue Fan, Kun Zhu, Yiqi Wang, Naisheng Zhang, Yunhe Fu, Xiaoyu Hu

**Affiliations:** Department of Clinical Veterinary Medicine, College of Veterinary Medicine, Jilin University, Changchun, 130062 Jilin Province China

**Keywords:** Calcium-ROS-AMPK-mTOR-autophagy pathway, Gut-mammary axis, Mastitis, *S. maltophilia*

## Abstract

**Background:**

Mastitis is an inflammatory disease of the mammary gland that has serious economic impacts on the dairy industry and endangers food safety. Our previous study found that the body has a gut/rumen-mammary gland axis and that disturbance of the gut/rumen microbiota could result in ‘gastroenterogenic mastitis’. However, the mechanism has not been fully clarified. Recently, we found that long-term feeding of a high-concentrate diet induced mastitis in dairy cows, and the abundance of *Stenotrophomonas maltophilia* (*S. maltophilia*) was significantly increased in both the rumen and milk microbiota. Accordingly, we hypothesized that ‘gastroenterogenic mastitis’ can be induced by the migration of endogenous gut bacteria to the mammary gland. Therefore, this study investigated the mechanism by which enterogenic *S. maltophilia* induces mastitis.

**Results:**

First, *S. maltophilia* was labelled with superfolder GFP and administered to mice via gavage. The results showed that treatment with *S. maltophilia* promoted the occurrence of mastitis and increased the permeability of the blood-milk barrier, leading to intestinal inflammation and intestinal leakage. Furthermore, tracking of ingested *S. maltophilia* revealed that *S. maltophilia* could migrate from the gut to the mammary gland and induce mastitis. Subsequently, mammary gland transcriptome analysis showed that the calcium and AMPK signalling pathways were significantly upregulated in mice treated with *S. maltophilia*. Then, using mouse mammary epithelial cells (MMECs), we verified that *S. maltophilia* induces mastitis through activation of the calcium-ROS-AMPK-mTOR-autophagy pathway.

**Conclusions:**

In conclusion, the results showed that enterogenic *S. maltophilia* could migrate from the gut to the mammary gland via the gut-mammary axis and activate the calcium-ROS-AMPK-mTOR-autophagy pathway to induce mastitis. Targeting the gut-mammary gland axis may also be an effective method to treat mastitis.

**Supplementary Information:**

The online version contains supplementary material available at 10.1186/s40104-023-00952-y.

## Introduction

Mastitis is one of the most serious diseases of dairy cows, causing huge economic losses in animal husbandry and posing a serious threat to human health [1]. According to statistics, mastitis occurs in up to 50% of dairy cows worldwide, and the cure rate is limited [2]. The aetiology of mastitis in dairy cattle is complex, and it is commonly believed that the major cause of mastitis is the invasion of exogenous microbial pathogens, such as *Staphylococcus aureus* and *Escherichia coli* [3]*.* However, recent studies have found that in addition to mammary gland infection by exogenous pathogenic microorganisms, endogenous pathogenic bacteria from the gut microbiota may also be infectious agents [4–7]. Evidence has shown that there are significant differences in the structure, abundance, and function of gut microbiota between cows with mastitis and healthy cows and that mastitis is induced in germ-free mice transplanted with faeces from cows with mastitis [8]. Our previous study showed that antibiotic-induced alterations in the gut microbiota in mice disrupted the blood-milk barrier and increased susceptibility to mastitis [6]. In addition, we previously showed that a subacute rumen acidosis (SARA) model in dairy cows induced by feeding a high-concentrate diet promoted mastitis symptoms in the mammary gland and increased the permeability of the blood-milk barrier and the gut barrier. In addition, *Stenotrophomonas* abundance was increased in both the rumen and milk of SARA cows, and mastitis was induced in lactating mice by oral administration of *S. maltophilia* [4]. These results indicate that enterogenic *S. maltophilia* is an important factor in inducing mastitis, but its pathogenic mechanism remains unclear.

Based on the concepts of the "gut-brain axis", the "gut-liver axis", the "gut-lung axis", and the "gut-kidney axis", numerous studies have shown that the gut microbiota not only affects the gut but also affects the occurrence and development of diseases in other organs, such as the brain, liver, lung and kidney, through the migration of bacteria and their metabolites in blood or lymph circulation [9–11]. Recent studies have also reported that gut bacteria can migrate to the mammary gland, especially lactating mammary gland tissues, through endogenous pathways, such as the blood or lymphatic circulation [12, 13]. Yong et al. [14] found that *Ruminococcus*, *Bifidobacterium* and *Peptostreptococcaceae* coexisted in the milk, blood and faeces of the same individual dairy cows. This finding suggested that gut bacteria can migrate to the mammary gland through an endogenous pathway in lactating cows. In addition, Jeon et al. [15] found that there were pathogenic bacteria in the blood after delivery, which could migrate to the uterus and induce endometritis. Therefore, we hypothesized that *S. maltophilia*, a pathogenic bacterium implicated in mastitis, may induce mastitis by migrating from the gut to the mammary gland.

To determine the location of the migration of *S. maltophilia*, superfolder green fluorescent protein (sf-GFP) was selected for labelling. Green fluorescent protein (GFP) is a protein composed of approximately 238 amino acids that can be excited by both blue and ultraviolet light, emitting green fluorescence. Through genetic engineering technology, the GFP gene can be transferred into the genomes of different species and continuously expressed in offspring. Sf-GFP is a well-designed folded version of GFP that exhibits greater resistance to chemical denaturants and extreme temperatures. Therefore, in this study, we tried to explore the mechanism of mastitis induced by enterogenic *S. maltophilia.*

## Materials and methods

### Animals

Twenty-four BALB/c female mice aged 8–12 weeks and 12 male mice were purchased from Liaoning Changsheng Biotechnology Co., Ltd. (Liaoning, China). Two females and one male were maintained in the same cage, and the mice had free access to food and water. After the female mice became pregnant, the male mice were removed. Female mice were used in the experiment one week after delivery. The experiment was carried out in accordance with the Guidelines for Experimental Animal Care and Use of Jilin University and was approved by the Animal Ethics Committee of Jilin University (20170318).

### Bacterial cultures

*S. maltophilia* was cultured in Luria–Bertani (LB) (HB0129, Qingdao Hope Bio-Technology, Shandong, China) medium at 37 °C for 24 h. The culture was incubated in an oscillating incubator at 180 r/min in an aerobic environment. The cultured *S. maltophilia* solution was centrifuged at 4 °C and 1,500 r/min for 5 min to concentrate the bacterial solution. Finally, the bacterial suspension was stored at 4 °C.

### Animal treatment

One week after delivery, 12 female mice were orally administered 10^10^ CFU *S. maltophilia*. The blood, mammary gland, mammary gland lymph node, mesenteric lymph node, spleen, lung and serum of the mice were collected in sterile centrifuge tubes after continuous gavage for 7 d.

### Haematoxylin-eosin (H&E) staining

The collected mammary gland and gut tissues of mice were fixed in 4% formaldehyde solution. The tissue was then embedded in paraffin and cut into 5-μm thick sections using a microtome (RM2245, Leica Biosystems, Wetzlar, Germany). The sections were stained with H&E, and histological analysis was performed using an optical microscope (Olympus, Tokyo, Japan). Finally, the pathological score of mammary gland tissue was determined based on previous studies [16].

### Immunohistochemistry (IHC)

The paraffin sections of the gut were submerged in xylene and a concentration gradient of ethanol for dewaxing and hydration. After antigen repair, a ready-to-use immunohistochemical hypersensitivity kit (KIT-9710, Maxin Biotechnologies, Fuzhou, China) was used according to the manufacturer's instructions. Then, the samples were incubated with ZO-1 and Claudin-3-specific primary antibodies (AF5145, AF0129, Melbourne, Australia) at 4 °C overnight. The next day, the samples were washed three times in PBS, and the kit was used for the subsequent procedures. The enzyme substrate chromogenic agent (DAB-031, Maxin Biotechonologies, Fuzhou, China) was used to develop the colour of the sections. Finally, haematoxylin was used to stain the nucleus and neutral resin was used for sealing.

### FITC-albumin assay

Paraffin sections of the gut were submerged in xylene and a concentration gradient of ethanol for dewaxing and hydration. After antigen repair, 5% goat serum (16210064, Thermo Fisher Scientific, Massachusetts, USA) was used for blocking. Samples were incubated in FITC solution (1.5 mg/mL) overnight at 4 °C. FITC (HY-66019) was bought from MedChemExpress (New Jersey, USA). After two washes in PBS (pH = 7.4) containing 1% Tween-20 (T104863, Shanghai Aladdin Biochemical Technology, China), the samples were stained with DAPI (C1005, Beyotime Biotechnology, Shanghai, China) in the dark for 3–5 min and glycerin (G8190, Beijing Solarbio Science & Technology, China) was used for sealing.

### Myeloperoxidase (MPO) activity determination

MPO activity in the mammary gland was determined using a myeloperoxidase assay kit (A044-1-1, Nanjing Jiancheng Bioengineering Institute, Jiangsu, China). Mammary gland tissues were processed according to the manufacturer’s instructions.

### Determination of inflammatory cytokine levels by ELISA

The levels of the proinflammatory cytokines TNF-α and IL-1β in tissues or cells were determined using ELISA kits (430204, 432604, BioLegend, California, USA). PBS was added to the mammary gland and gut tissues, which were subjected to homogenization. Then, the sediment was discarded, and the supernatant was retained for detection.

### Western blotting

Tissue protein extraction reagent (78510, Thermo Fisher Scientific) was added to tissues or cells to extract the total proteins, and the protein content was determined using a BCA Protein Assay Kit (23225, Thermo Fisher Scientific, California, USA). The protein samples were separated by 8%, 12%, and 15% SDS‒PAGE and then transferred to PVDF (IPVH00010, Thermo Fisher Scientific) membranes using the wet transfer method. After blocking at room temperature for 3 h with 5% skimmed milk powder, specific primary antibodies were added and samples were incubated at 4 °C overnight. After washing with TBST the next day, blots were incubated with murine or rabbit secondary antibodies for 2 h at room temperature. Finally, the membranes were washed again with TBST, and a Western blot detection system (Tanon 4500, Shanghai, China) was used to measure protein expression. The specific primary antibodies used in this experiment included β-actin (4967S), AMPK (2532S), p-AMPK (2535S), mTOR (2972S), p-mTOR (2971S), p65 (8242S), p-p65 (3033S), p38 (9212S), p-p38 (9211S), p62 (5114S), LC3 (2775S), GFP (ZY60501M), ZO-1 (AF5145), Occludin (DF7504), Claudin-3 (AF0129). Anti-GFP antibody were purchased from Zeye Biotechnology (Shanghai, China), ZO-1, Occludin and Claudin-3 were purchased from Affinity Biosciences, and all other antibodies were purchased from Cell Signaling Technology.

### Routine blood and blood biochemical examination

Blood (1 mL) was collected from each mouse by eyeball blood collection. A portion of the mouse blood sample was collected in EDTA anticoagulant blood collection tubes (SANLI industry, Hunan, China), and the other portion was collected in sterile centrifuge tubes. The blood in the EDTA anticoagulant blood collection tube was used for routine blood examination. The blood in the sterile centrifuge tube was centrifuged at 4 °C and 1,500 r/min for 10 min to obtain serum for blood biochemical examination.

### Superfolder-GFP fluorescent labelling of *S. maltophilia*

*S. maltophilia* was inoculated in media containing different types of antibiotics overnight to observe the growth of the strain in order to select the appropriate antibiotic to mark plasmid resistance. *S. maltophilia* was inoculated into 5 mL LB liquid medium and transferred to 50 mL LB liquid medium at a ratio of 1:100 the next day. When the OD grew to approximately 0.8, the bacteria were centrifuged and collected. The bacteriosome was washed three times with 10% glycerol, and finally, the bacteriosome was resuspended in 2 mL of 10% glycerol to prepare the receptor cells. Five microlitres of green fluorescent plasmid was added to 90 μL of prepared competent cells and the cells were incubated on ice for 5 min, electrotransformed at 2,500 KV, coated on the resistant plate, and cultured overnight at 37 °C. In addition, according to the sequence of sf-GFP, Primer 5.0 software was used to design primers, and PCR amplification and agarose gel electrophoresis were carried out to verify whether the plasmid was successfully transferred. Finally, we determined whether the bacterial solution emits green fluorescence under 470 nm blue light.

### Bacterial load assay

One hundred microlitres of sterile PBS was added to every 0.01 g of mammary gland tissue, and the tissues were placed into a rapid tissue grinder for complete homogenization. Then, the tissue homogenate was uniformly coated on LB solid medium containing 34 μg/mL chloramphenicol (C8050, Beijing Solarbio Science & Technology, China). After the solid plate was placed into a 37 °C incubator for 24 h, the colonies on the plate were counted.

### Agarose gel electrophoresis

Primers were designed using Primer 5.0 software according to the sequence of sf-GFP gene fragments (Table S1). DNA from the mammary gland, mammary gland lymph node, mesenteric lymph node, spleen, lung and serum was extracted using the blood/cell/tissue genomic DNA extraction kit. PCR amplification was performed using a 25-μL system, including 12.5 μL DNA polymerase (D7053S, Beyotime Biotechnology, Shanghai, China), 7.5 µL ddH_2_O, 3 μL DNA and 1 µL upstream and downstream primers. The mixed samples were PCR amplified in a C1000 Touch PCR (Bio-Rad, California, USA) instrument. The reaction conditions were 95 °C for 5 min; 35 cycles at 95 °C for 30 s, annealing at 58 °C for 30 s, and 72 °C for 30 s; and a final extension at 72 °C for 5 min. The samples were electrophoretically separated on a 1.2% agarose gel at 110 V for 30 min. Finally, the agarose gel was imaged using a Gel Doc XR+ System (Bio-Rad).

### Observation of bacteria under 470 nm blue light and 488 nm green light

The cell tissues were homogenized and evenly coated on LB solid medium containing 34 μg/mL chloramphenicol. Single colonies were selected after 24 h and incubated in LB liquid medium containing 34 μg/mL chloramphenicol at 37 °C and 180 r/min for 24 h. After the obtained bacterial suspension was added dropwise to the slides, the slides were sealed using glycerol. In addition, plain paraffin sections of mammary gland tissue and paraffin sections of 10 μg/mL DAPI-stained cell nuclei were used for observation. The samples were observed by confocal microscopy (Olympus, Tokyo, Japan) under blue light at 470 nm and green light at 488 nm.

### Transcriptome analysis of the mammary gland

According to the manufacturer’s instructions, TRIzol (15596018, Thermo Fisher Scientific) was used to purify RNA from a total of 8 samples from the control group and the maltose streptococcus group. A NanoDrop ND-1000 (NanoDrop, Wilmington, DE, USA) was used to assess the quantity and purity of total RNA, and a Bioanalyzer 2100 (Agilent, California, USA) was used to determine the integrity of RNA. The RNA samples that met the following criteria were employed in subsequent experiments: concentration > 50 ng/μL, RIN value >7.0, and total RNA > 1 μg; these samples satisfied the requirements needed for downstream experiments. PolyA (polyadenylate) RNA was specifically captured by two rounds of purification using oligo(dT) magnetic beads (Dynabeads Oligo (dT), cat. 25-61005, Thermo Fisher Scientific). After obtaining the downstream sequencing data, we filtered the downstream data to obtain high-quality sequencing data (clean data). The captured mRNA was fragmented under high temperature conditions using a magnesium ion interruption kit (NEBNextR Magnesium RNA Fragmentation Module, cat. E6150S, USA) at 94 °C for 5–7 min. The fragmented RNA was synthesized into cDNA by reverse transcriptase (Invitrogen SuperScriptTM II Reverse Transcriptase, cat. 1896649, CA, USA). These DNA‒RNA complex duplexes were then converted into DNA duplexes using *E. coli* DNA polymerase I (NEB, cat. m0209, USA) and RNase H (NEB, cat. m0297, USA), while the duplexes were doped with dUTP Solution (R0133, Thermo Fisher Scientific, California, USA), which complements the ends of the double-stranded DNA with flat ends. An A base was then added to each end to enable ligation to a splice with a T base at the end, and the fragment size was screened and purified using magnetic beads. The second strand was digested with UDG enzyme (NEB, cat.m0280, MA, US), then held by PCR-predenaturing at 95 °C for 3 min, denatured at 98 °C for a total of 8 cycles of 15 s each, annealed at 60 °C for 15 s, extended at 72 °C for 30 s, and the final extension was performed at 72 °C for 5 min to form a fragment size of 300 ± 50 bp library (strand-specific library). Finally, we performed double-end sequencing using Illumina NovaSeqTM 6000 (LC Bio Technology Co., Ltd., Hangzhou, Zhejiang, China) in PE150 sequencing mode according to standard practice. After obtaining the sequencing downstream data, we first filtered the downstream data to obtain high-quality sequencing data (clean data) and compared the high-quality sequencing data to the reference genome of this project species; we then performed analyses such as gene expression quantification, GSEA, gene difference analysis, and enrichment analysis. Data quality control and RNA-seq quality control were completed through FastQC (0.10.1) and RSeQC (4.0.0). Genome alignment was completed through Hisat2 (2.1.1). The splicing and merging of transcripts were completed through StringTie (2.1.6). Transcript quantification, differential comparison, and visualization were completed through DESeq2 (NA).

### Cell cultures and treatment

Mouse mammary epithelial cells (MMECs) were cultured in DMEM (SH30081.FS, HyClone, Utah, USA) containing 10% foetal bovine serum (FBS) and 1% penicillin and streptomycin (Hyclone, SV30010, Utah, USA). The cell abundance was allowed to reach 80%–90%, and the cells were transferred to 6-well plates for culture. When the cell abundance in the 6-well plate reached approximately 70%, the old medium was discarded, and 2 mL of DMEM containing 10% FBS and 6 × 10^5^ CFU/mL *S. maltophilia* solution (1, 2 and 4 μL) was added to each well. In addition, 3 mmol/L of the calcium chelator EGTA (HY-D0861, MedChemExpress, New Jersey, USA) and 2.5 μmol/L of the autophagy inhibitor 3-MA (HY-19312, MedChemExpress, New Jersey, USA) were also used for cell treatment [17, 18].

### ROS determination

MMECs were cultured in confocal Petri dishes and then treated with C11-BODIPY 581/591 (10 μmol/L) for 30 min. The cells were washed with PBS three times for 5 min each time. ROS levels were measured using confocal microscopy at 594 and 488 nm light (Olympus, Tokyo, Japan).

### Ca^2+^ ion level measurement

MMECs were cultured in confocal Petri dishes and then treated with 5 μmol/L Fluo-4 AM (Invitrogen, Thermo Fisher Scientific) for 30 min [19]. The cells were washed with PBS three times for 5 min each time. Ca^2+^ ion levels were measured using confocal microscopy at 594 and 488 nm (Olympus, Tokyo, Japan).

### Statistical analysis

GraphPad Prism 8.0 was used for data analysis and to generate figures. The data are reported as the standard deviation (SD) of the sum of the results of a representative experiment. The experiments in this paper were performed with three independent replicates. Significant differences were noted when *P* < 0.05.

## Results

### Mastitis was induced in mice by oral gavage of sf-GFP-*S. maltophilia*

The plasmids used to label *S. maltophilia* are shown in Fig. S1A, and the sf-GFP fragment was 5,855 bp in length. PCR amplification and agarose gel electrophoresis were performed on the plasmid-transformed colonies. Agarose gel analysis revealed 717-bp-long products (Fig. S1B). This result indicates that the plasmid was successfully transferred into *S. maltophilia*. The green fluorescence of the sf-GFP-labelled *S. maltophilia* solution was observed under 470 nm blue light (Fig. S1C).

**Fig. 1:**
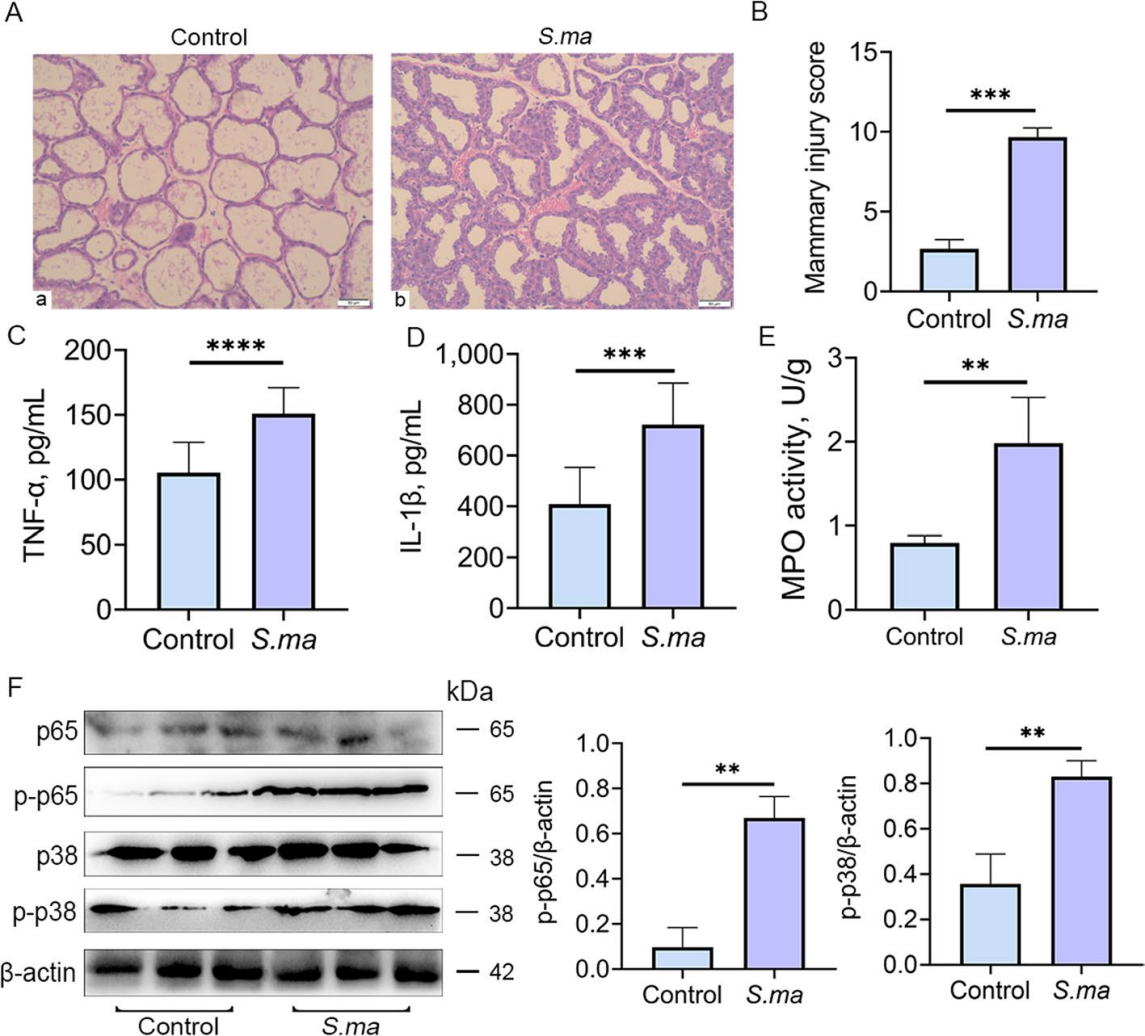
Mastitis was induced by oral gavage of sf-GFP-*S. maltophilia* in mice. After the mice were treated with 10^10^ CFU *S. maltophilia* for one week, the mammary gland tissues were collected and their inflammatory indicators were detected. **A** H&E staining of mammary tissue in control and *S. maltophilia* gavage mice. The *S. maltophilia* gavage group showed incomplete mammary gland follicular structure and inflammatory cell infiltration. **B** Histopathological scores of mammary gland tissues in control and *S. maltophilia* gavage mice. **C **and **D** Expression of inflammatory cytokines TNF-α and IL-1β in mammary gland tissues. **E** MPO activity in mammary tissues. **F** Significant activation of p-38/MAPK and NF-κB signaling pathways and significant upregulation of p-p38, p-p65 protein expression in mammary tissue of mice after *S. maltophilia* gavage. The data are presented as mean ± SD. One-way analysis of variance was performed for statistical analysis. ^*^P < 0.05, ^**^P < 0.01 and ^***^P < 0.001 indicate significance from each group. ns, no significance

After treatment with sf-GFP-*S. maltophilia,* the mammary glands of all mice exhibited varying degrees of inflammation and significant pathological features of acinar structure damage and inflammatory cell infiltration (Fig. 1A) and increased pathological scores compared to the control group mice (Fig. 1B). In addition, the levels of the proinflammatory cytokines TNF-α and IL-1β and MPO activity in the mammary glands of the gavage group were significantly increased compared with those of the control group (Fig. 1C–E). Moreover, p-p38 and p-p65 protein expression was significantly upregulated in the mammary glands of mice in the oral gavage group (Fig. 1F). These results demonstrate that oral gavage of *S. maltophilia* can induce mastitis in mice.

### Treatment with sf-GFP-*S. maltophilia* disrupts the integrity of the blood-milk barrier

**Fig. 2:**
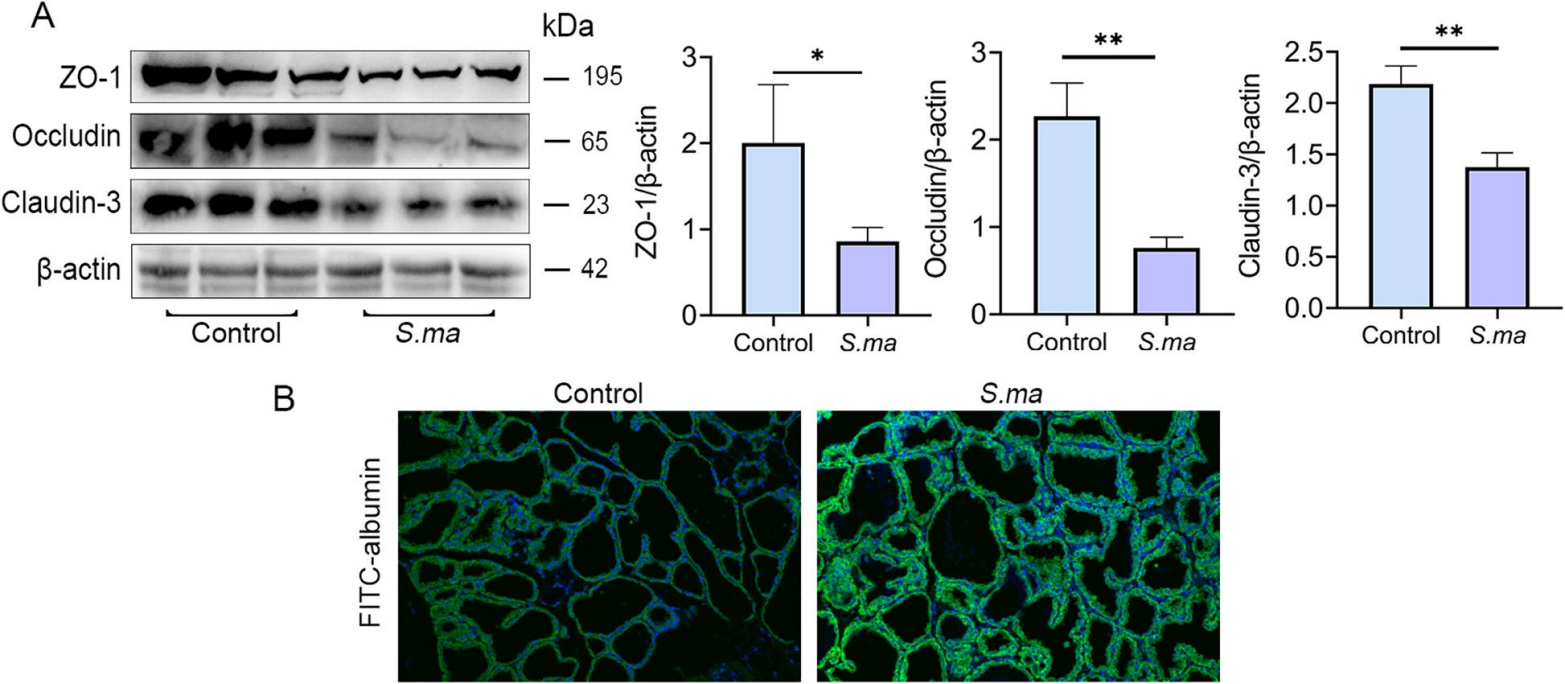
Treatment with sf-GFP-*S. maltophili*a destroys the integrity of the blood-milk barrier. After the mice were treated with 10^10^ CFU *S. maltophilia* for a week, mammary gland tissues were collected and the integrity of blood-milk barrier was detected. **A** Expression of tight junction proteins ZO-1, Occludin, Claudin-3 in mouse mammary gland. **B** FITC-albumin fluorescence staining of mammary gland tissue to determine the degree of albumin infiltration. The data are presented as mean ± SD. One-way analysis of variance was performed for statistical analysis. ^*^P < 0.05, ^**^P < 0.01 and ^***^P < 0.001 indicate significance from each group. ns, no significance

The integrity of the blood-milk barrier was evaluated after oral gavage of *S. maltophilia*. The results showed that the expression of the tight junction proteins ZO-1, Occludin, and Claudin-3 in the mammary glands of mice in the *S. maltophilia*-treated group was significantly reduced compared with that in the mammary glands of mice in the control group (Fig. 2A). In addition, the location of albumin in the mammary gland was determined by FITC-albumin immunofluorescence staining, and the results showed that treatment with *S. maltophilia* induced leakage of FITC-albumin from the interstitial side into the alveolar lumen (Fig. 2B). These results suggested that *S. maltophilia* increased the permeability of the blood-milk barrier in mice.

### Treatment with *S. maltophilia* induced inflammation in the gut and disrupted the gut barrier

**Fig. 3:**
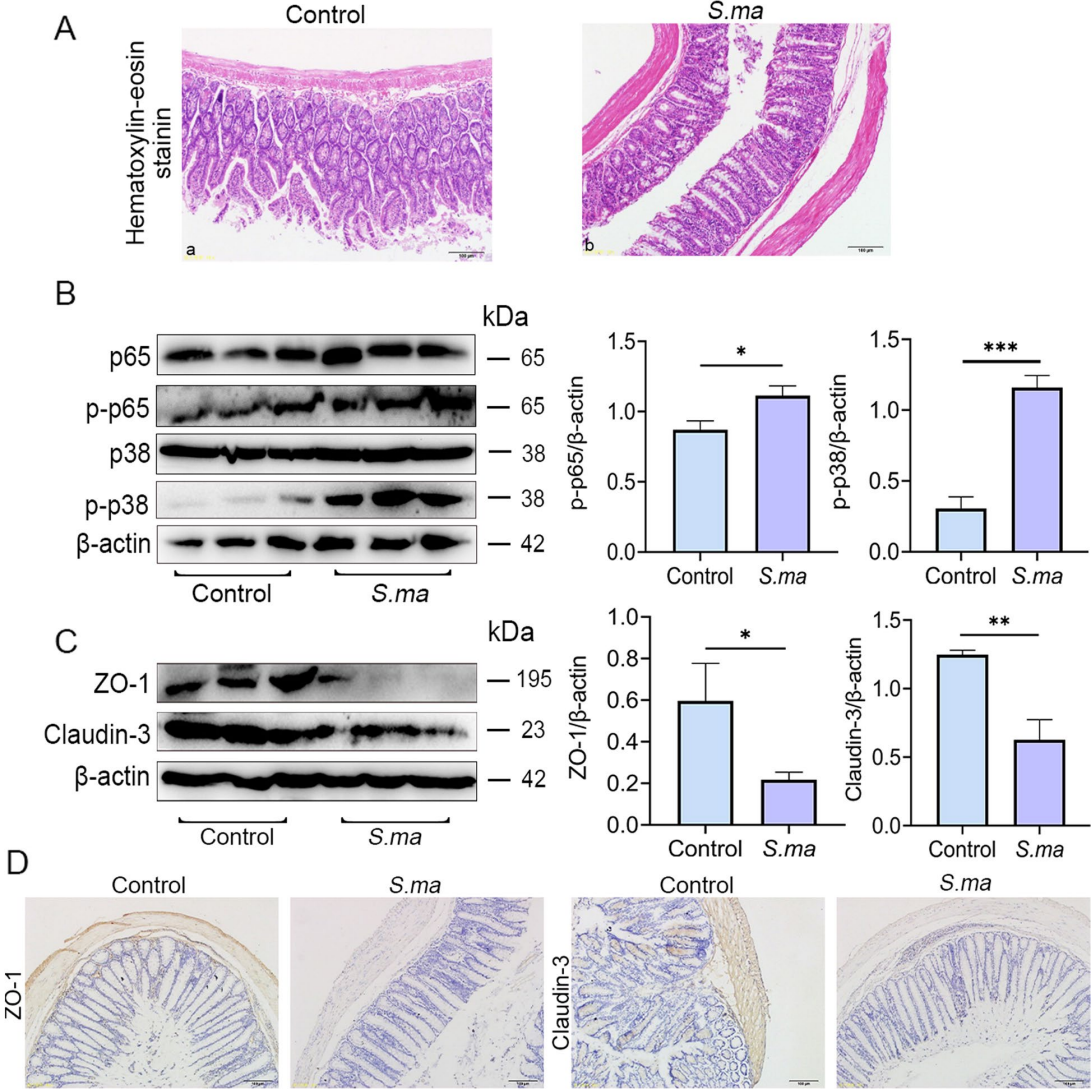
Treatment with *S. maltophilia* induced inflammation in the gut and destroyed the gut barrier. After the mice were treated with 10^10^ CFU *S. maltophilia* for a week, the gut tissues were collected and their inflammatory indicators were detected. At the same time, the integrity of gut barrier was also detected. **A** Gut H&E sections of control and *S. maltophilia* gavage mice, *S. maltophilia* group showing neutrophil, lymphocyte and plasma cell infiltration and altered crypt structure. **B** Intestinal p38/MAPK and NF-κB signaling pathway levels in mice in the untreated and oligotrophic Aeromonas gavage groups, with p-p65 and p-p38 significantly upregulated. **C** Changes in the expression of tight junction proteins ZO-1 and Claudin-3 in mouse gut tissues after *S. maltophilia* gavage. **D** Immunohistochemistry of colonic tight junction protein ZO-1 and Claudin-3 in control and *S. maltophilia* gavage mice. The data are presented as mean ± SD. One-way analysis of variance was performed for statistical analysis. ^*^P < 0.05, ^**^P < 0.01 and ^***^P < 0.001 indicate significance from each group. ns, no significance

*S. maltophilia* also induced pathological damage to the gut, as demonstrated by the increase in the infiltration of neutrophils, lymphocytes and plasma cells, as well as changes in crypt structure, compared with the control group mice (Fig. 3A). In addition, the expression of components of the p-p-38/MAPK and p-p65-NF-κB signalling pathways was significantly increased in the gut tissues of mice in the gavage group (Fig. 3B). Furthermore, *S. maltophilia* also disrupted the integrity of the gut barrier, as demonstrated by the reduced expression of the tight junction proteins ZO-1 and Claudin-3 in mice in the gavage group compared with that in the control group (Fig. 3C and D).

### Ingested *S. maltophilia* migrates from the gut to the mammary gland

**Fig. 4:**
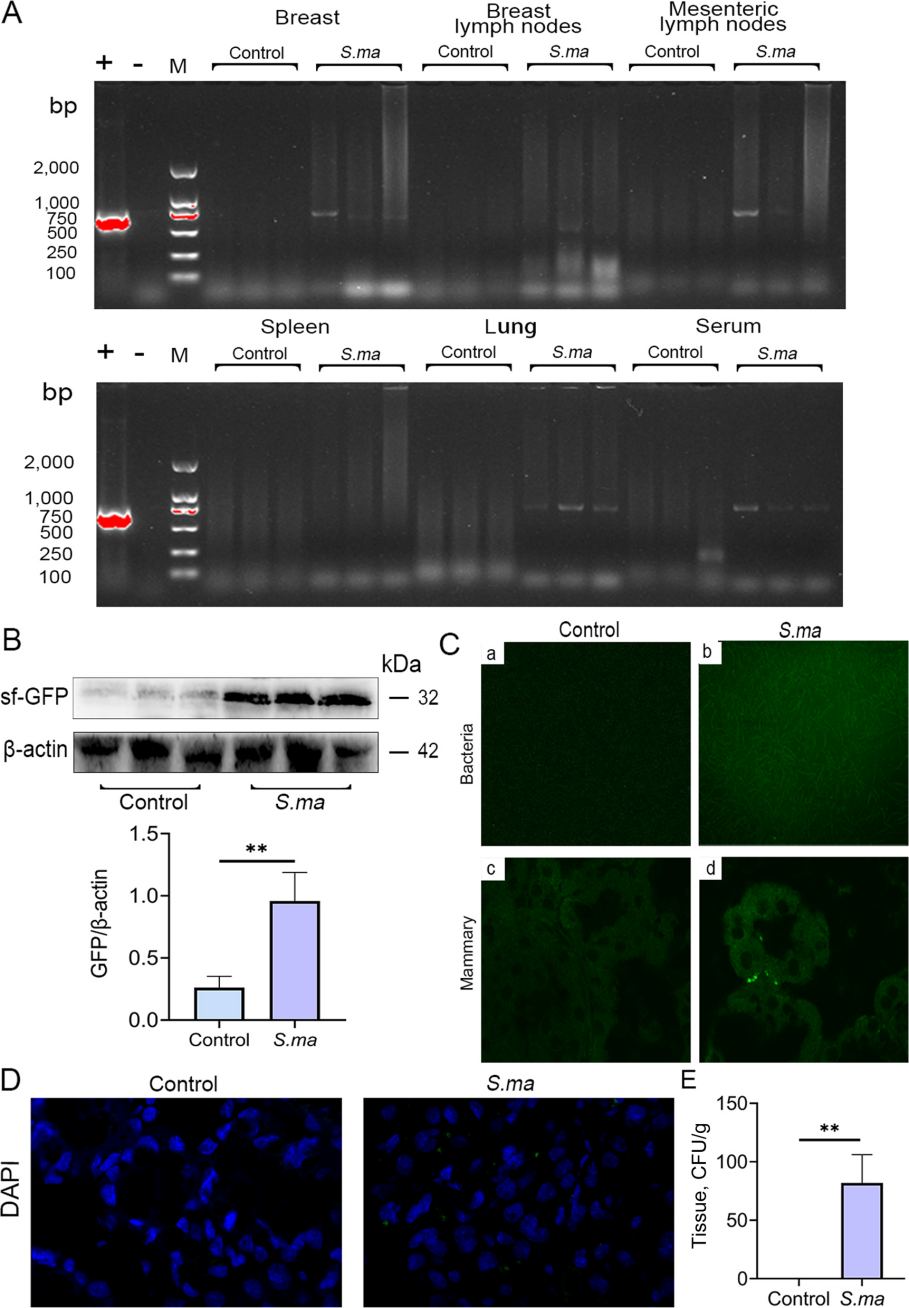
Ingested *S. maltophilia* migrates from the gut to the mammary gland. The *S. maltophilia* were labeled with sf-GFP protein and then gavaged to mice for a week. The organs and blood of the mice were collected and the bacterial location was tracked. **A** The presence of sf-GFP gene fragments in mammary gland, breast lymph node, mesenteric lymph node, spleen, lung and serum was identified by agarose gel. **B** Western blot was used to detect the expression of sf-GFP protein in mammary gland. **C** Observe whether there are green fluorescent bacteria in mammary culture and mammary paraffin section. **D** After DAPI staining the nuclei of paraffin sections of mammary gland, observe the bacteria that emit green fluorescence. **E** The abundance of *S. maltophilia* in the mammary gland of untreated group and gavage group. The data are presented as mean ± SD. One-way analysis of variance was performed for statistical analysis. ^*^P < 0.05, ^**^P < 0.01 and ^***^P < 0.001 indicate significance from each group. ns, no significance

To investigate whether mastitis is induced by enterogenic *S. maltophilia* migrating from the gut to the mammary gland through the disrupted gut and blood-milk barriers, we detected the expression of the sf-GFP gene fragment in *S. maltophilia*. Gene fragments of sf-GFP were found in the mammary gland, mammary lymph nodes, mesenteric lymph nodes, lungs and serum of mice (Fig. 4A). Western blot analysis also showed that a large amount of sf-GFP was present in the mammary glands of mice in the *S. maltophilia*-treated group (Fig. 4B). In addition, green fluorescence was observed in mammary gland tissue cultures and paraffin sections of the mammary gland in the gavage group (Fig. 4C). After nuclear staining with DAPI, the location of the bacteria was further determined (Fig. 4D). Finally, we detected the abundance of *S. maltophilia* in the mammary glands of mice in the different groups. The results showed significantly higher *S. maltophilia* levels in the gavage group than in the control group (Fig. 4E). Furthermore, increased white blood cells (WBCs), mean corpuscular haemoglobin concentration (MCHC), lymphocytes (LYM), neutrophils (GRAN), and monocytes (MONO) were noted in the gavage group compared with the control group (Table S2). In addition, significantly increased globulin (GLO), white cell ratio (A/G), alanine aminotransferase (ALT) and blood urea nitrogen (BUN) levels were noted in mice in the gavage group compared with the control group (Table S3). These results suggested that *S. maltophilia* can migrate from the gut to the mammary gland and induce a systemic inflammatory response in mice.

### Treatment with *S. maltophilia* altered immune and metabolic pathways in mammary gland tissues

**Fig. 5:**
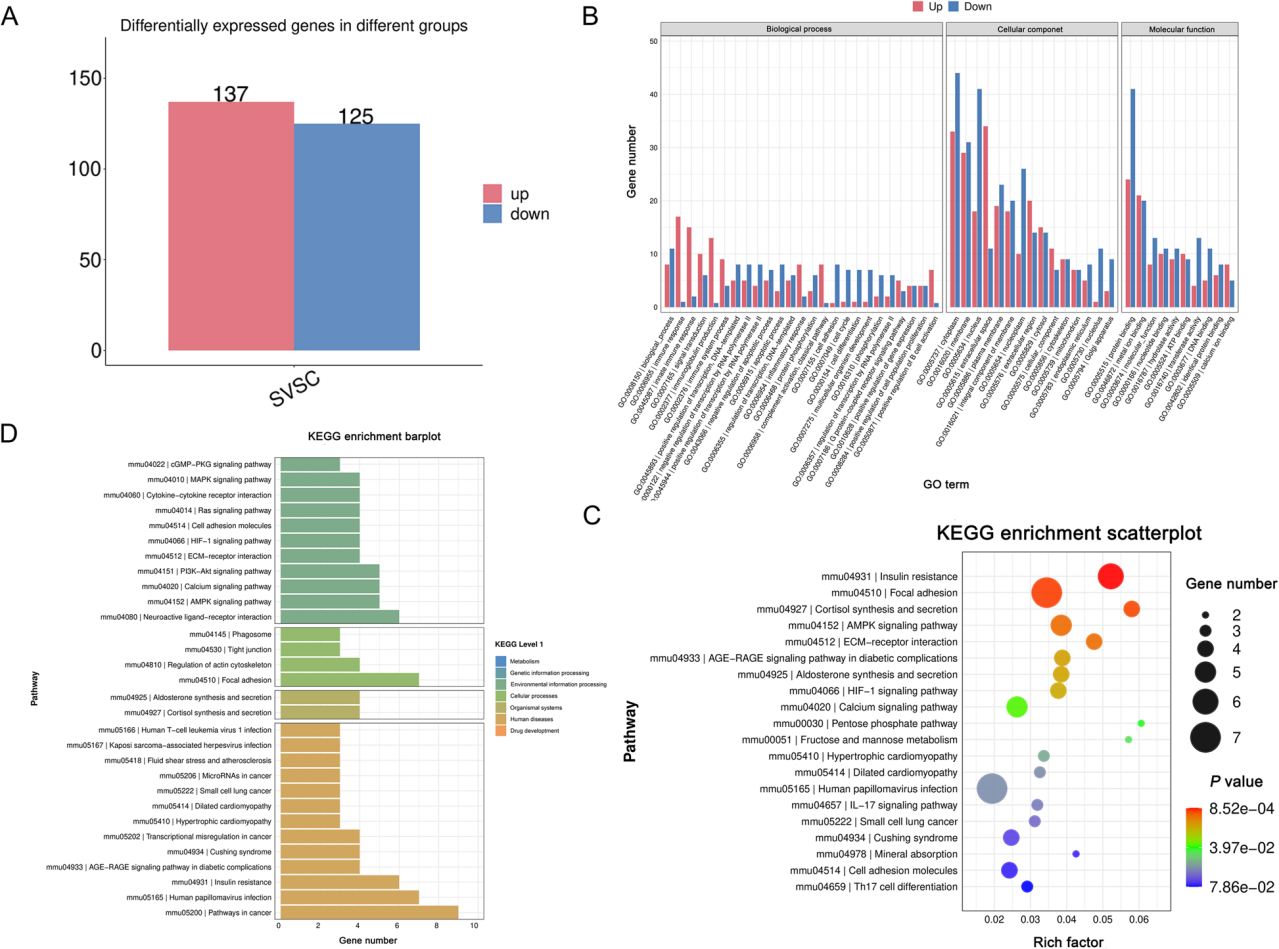
Treatment with *S. maltophilia* altered immune and metabolic pathways in the mammary gland tissues. Mammary gland tissues were collected for transcriptome sequencing after the mice were treated with 10^10^ CFU *S. maltophilia* for a week. **A** Statistics of up and down frequency of differentially expressed genes. The number of differentially expressed genes up regulated (log_2_FC ≥ 1 and *Q* < 0.05) and down regulated (log_2_FC ≤ −1 and *Q* < 0.05) in each comparison group was counted. **B** Up and down histogram of GO enrichment. The ordinate represents the number of differential genes enriched to GO entries, the number of up and down genes is red and blue respectively, and the abscissa represents GO entries. **C** In the KEGG enrichment bubble chart, the abscissa Rich Factor represents the proportion of the number of differential genes located in the path to the total number of genes located in the path, and the ordinate is the KEGG pathway. **D** KEGG level histogram: the abscissa is the number of differential genes included in the pathway, and the color represents the KEGG primary classification

Based on the transcriptome results, we conducted gene differential expression analysis and differentially expressed gene function and pathway analysis. Compared with the control group, 137 differentially upregulated genes and 125 downregulated genes were found in the *S. maltophilia* treatment group (Fig. 5A). Gene Ontology (GO) describes the molecular function (mf), cellular component (cc) and biological process (bp) of genes. As seen from the upper and lower histograms of GO enrichment, important biological processes, such as the immune response, the innate immune response and immunoglobulin production, were significantly upregulated in the *S. maltophilia* gavage group compared to the control group. The processes of cell adhesion, cell cycle and cell differentiation were significantly downregulated. In the cellular component category, the cytosol, nucleus and plasma membrane were significantly downregulated. In terms of molecular function, protein binding and molecular function were significantly downregulated (Fig. 5B). Kyoto Encyclopedia of Genes and Genomes (KEGG) analysis was used to assess the expression of signalling pathways. The KEGG bubble diagram revealed the top 20 enrichment pathways when compared with the control group, and a significant difference between AMPK and calcium signals was observed (Fig. 5C). In addition, the KEGG hierarchical histogram also showed significant differences in the MAPK signalling pathway, cytokine-receptor interactions, the calcium signalling pathway, the AMPK signalling pathway, and tight junction protein cellular processes (Fig. 5D). These results suggested that *S. maltophilia* induces mastitis by regulating calcium, the AMPK signalling pathway, and cell adhesion molecules.

### *S. maltophilia* significantly activated calcium, AMPK, mTOR, and autophagy signalling in MMECs

**Fig. 6:**
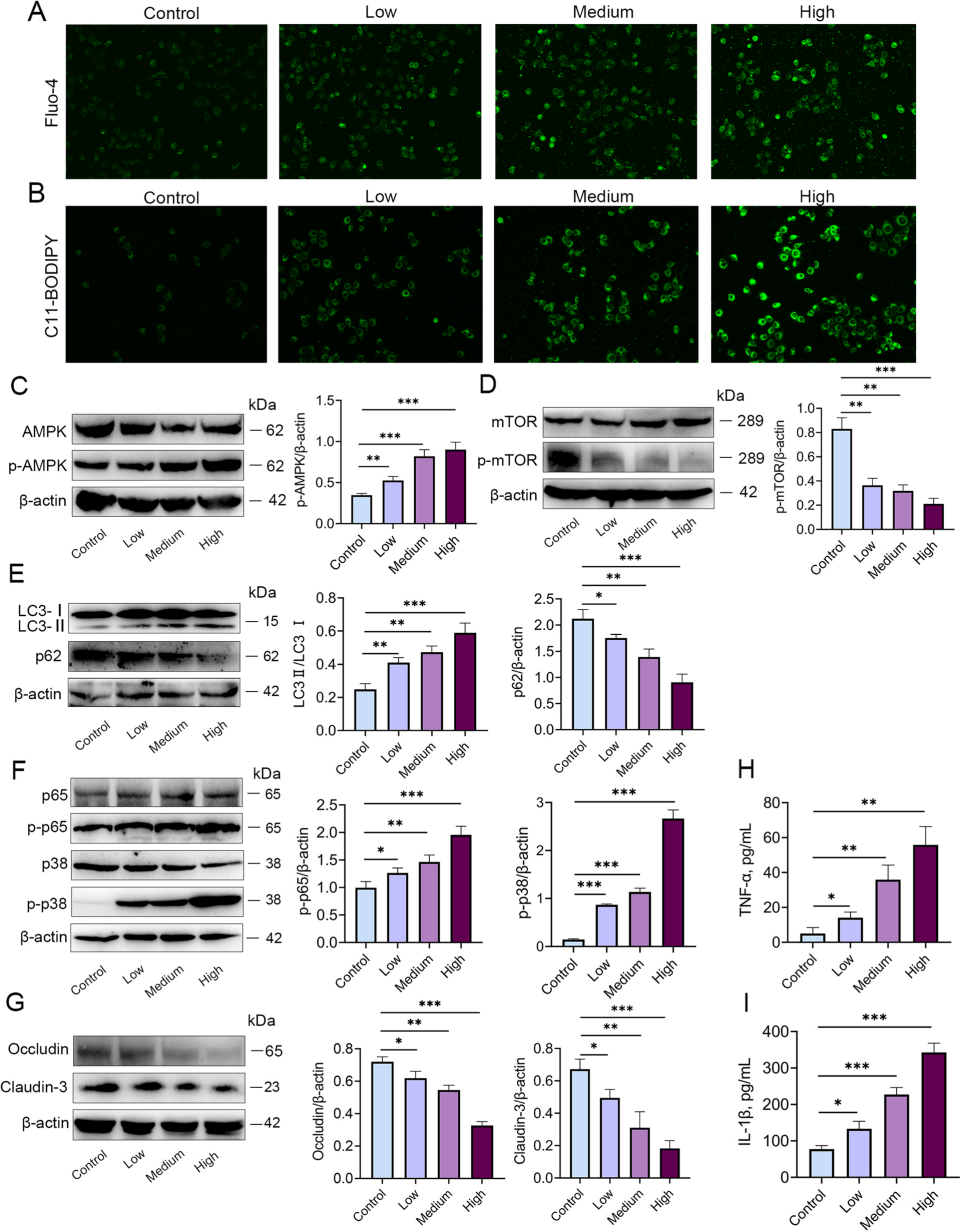
*S. maltophilia* significantly activated calcium, AMPK, mTOR, and autophagy signalling in MMECs. MMECs were collected after 6 h of treatment in a medium containing 3 × 10^2^, 6 × 10^2^, 1.2 × 10^2^ CFU/mL of *S. maltophilia*. **A** Calcium concentration was measured by Flou-4 fluorescence probe. **B** ROS level was measured by C11-BODIPY fluorescent probe. **C** Level changes of AMPK signaling pathway. The p-AMPK level was significantly increased. **D** Level changes mTOR signaling pathways in MMECs. The p-mTOR level was significantly decreased. **E** The autophagic level of MMECs was significantly increased by *S. maltophilia* treatment. **F** The expression of p-p38 and p-p65 in the p/38MAPK and NF-κB signal pathways of MMECs was significantly increased under the stimulation of *S. maltophilia* in a concentration gradient. **G** The tight junction proteins Occludin and Claudin-3 in MMECs were significantly reduced. **H** and **I** Expression of inflammatory cytokines TNF-α and IL-1β in MMECs. The data are presented as mean ± SD. One-way analysis of variance was performed for statistical analysis. ^*^P < 0.05, ^**^P < 0.01 and ^***^P< 0.001 indicate significance from each group. ns, no significance

Based on the transcriptome results, we treated MMECs with different concentrations of *S. maltophilia* to assess calcium and AMPK signalling pathway activation. The results showed that calcium ion levels and p-AMPK expression were significantly increased in response to *S. maltophilia* infection (Fig. 6A–C). Studies have shown that an increase in calcium is associated with AMPK/mTOR/autophagy signalling pathway activation [20]. In addition, we also detected the levels of ROS, the expression of mTOR, autophagy, blood-milk barrier tight junction proteins, and the inflammatory signalling NF-κB and MAPK pathways. The results showed that *S. maltophilia* dose-dependently reduced the expression of p-mTOR and the p62 protein, which are involved in autophagic signalling, whereas the levels of LC3 II and ROS were significantly increased (Fig. 6D and E). In addition, the expression of p-p38-MAPK and p-p65-NF-κB was increased, whereas the levels of the tight junction proteins Occludin and Claudin-3 were significantly reduced in mice treated with *S. maltophilia* (Fig. 6F and G). Furthermore, the concentrations of the proinflammatory cytokines TNF-α and IL-1β were increased after treatment with *S. maltophilia* (Fig. 6H and I). These results suggested that mastitis induced by *S. maltophilia* was associated with the activation of calcium/ROS/AMPK/mTOR/autophagy and the NF-κB and MAPK signalling pathways.

### Inhibition of calcium ions reduces the inflammatory response by regulating the activation of the ROS/AMPK/mTOR/autophagy pathway induced by *S. maltophilia*

**Fig. 7:**
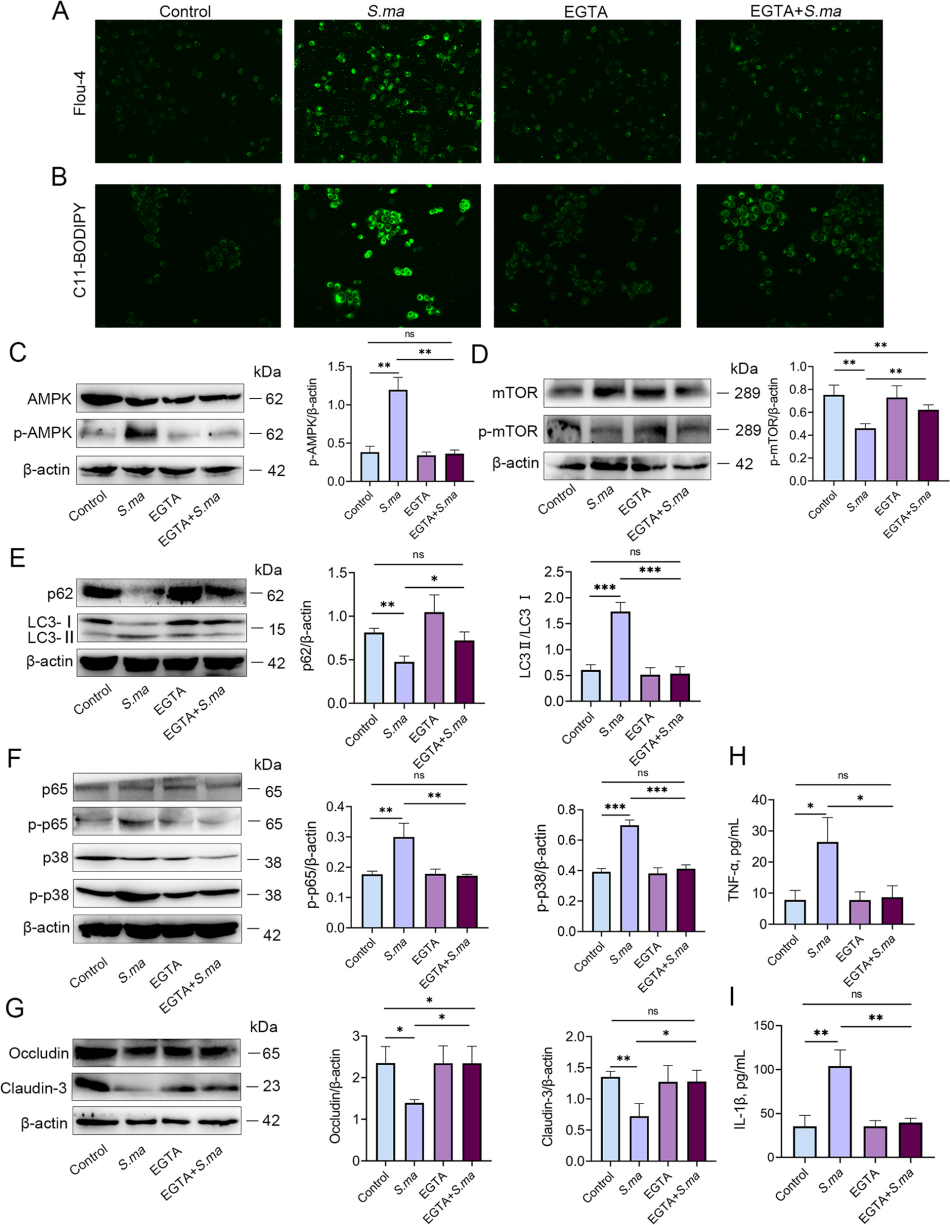
Inhibition of calcium ions reduces the inflammatory response by regulating the ROS/AMPK/mTOR/autophagy pathway induced by *S. maltophilia*. MMEC was pretreated with calcium chelating agent EGTA, and then added with *S. maltophilia* for 6 h before being collected. **A** Calcium concentration was measured by Flou-4 fluorescence probe. **B** ROS level was measured by C11-BODIPY fluorescent probe. **C** and **D** Level changes of AMPK and mTOR signaling pathways in MMEC. The increase of p-AMPK level and the decrease of p-mTOR level were alleviated by EGTA. **E** The autophagic level of MMECs was significantly reduced by EGTA pretreatment. **F** Under EGTA pretreatment, the expression of p-p38 and p-p65 in the p/38MAPK and NF-κB signaling pathways of MMEC was significantly alleviated. **G** The decrease of tight junction proteins Occludin and Claudin-3 in MMEC was alleviated. **H**–**I** Expression of inflammatory cytokines TNF-α and IL-1β in MMECs. The data are presented as mean ± SD. One-way analysis of variance was performed for statistical analysis. ^*^P < 0.05, ^**^P < 0.01 and ^***^P < 0.001 indicate significance from each group. ns, no significance

**Fig. 8:**
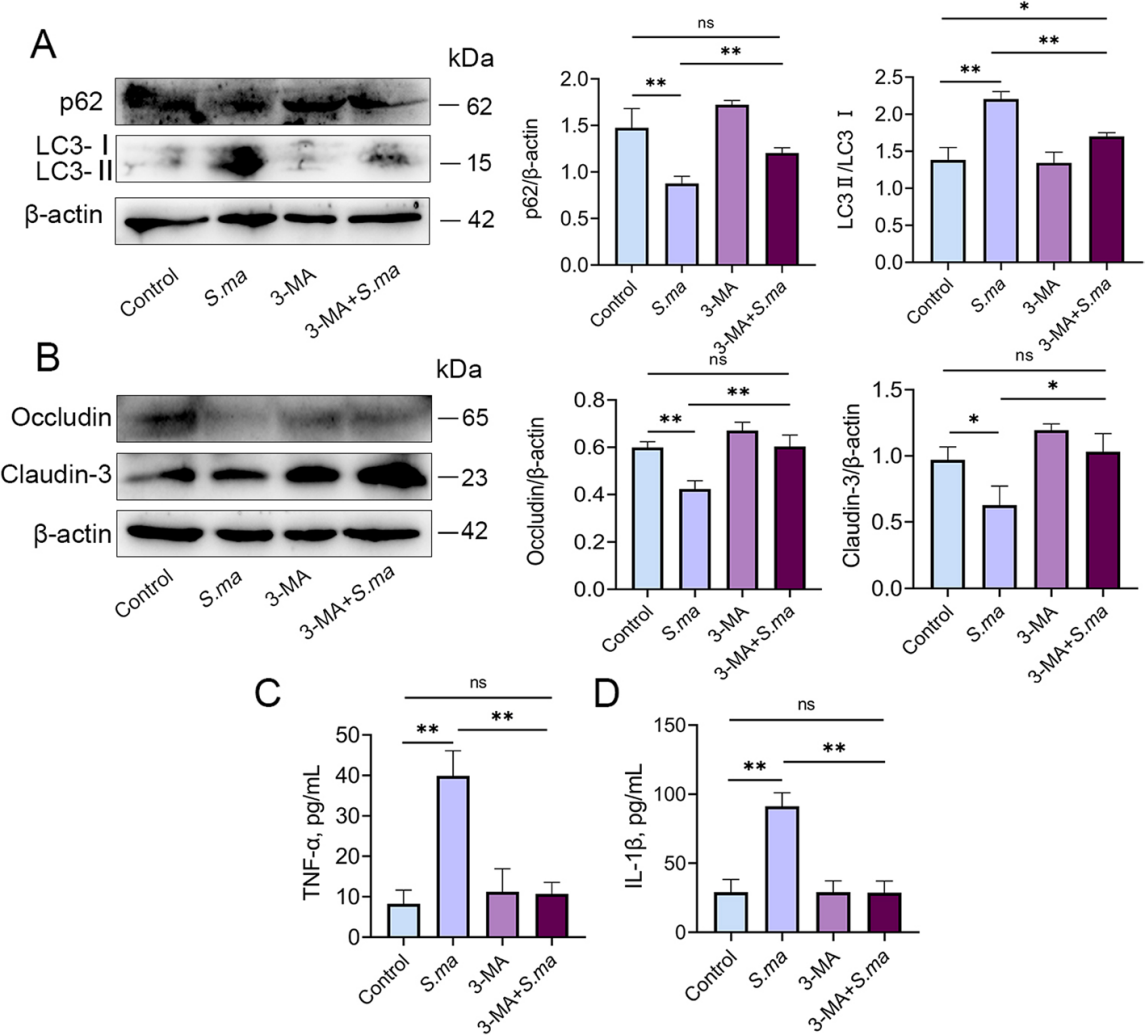
Inhibition of autophagy alleviates the inflammatory response induced by *S. maltophilia*. MMECs were pretreated with autophagy inhibitor 3-MA, and then added with *S. maltophilia* for 6 h before being collected. **A** The autophagic level of MMECs was significantly reduced by 3-MA pretreatment. The decrease of p62 and the increase of LC3 II were significantly alleviated. **B** The destruction of the tight junction proteins Occludin and Claudin-3 in MMECs was significantly alleviated by 3-MA pretreatment. **C** and **D** Expression of inflammatory cytokines TNF-α and IL-1β in MMECs. The data are presented as mean ± SD. One-way analysis of variance was performed for statistical analysis. ^*^P < 0.05, ^**^P < 0.01 and ^***^P < 0.001 indicate significance from each group. ns, no significance

To verify whether the calcium signalling pathway acts as an upstream target of *S. maltophilia* to induce mastitis and the disruption of tight junction proteins, MMECs were pretreated with the calcium chelator EGTA. As shown in Fig. 7A and B, EGTA inhibited the increased levels of calcium ions and ROS induced by *S. maltophilia*. Moreover, inhibition of calcium signalling reversed the upregulation of p-AMPK and downregulation of p-mTOR by *S. maltophilia* (Fig. 7C and D). Additionally, EGTA treatment alleviated the increase in the level of autophagy induced by *S. maltophilia*. The decrease of p62 and the increase of LC3 in MMECs were both alleviated (Fig. 7E). In addition, the expression of p-p38 and p-p65 was decreased (Fig. 7F), and the expression of Occludin and Claudin-3 was increased after inhibition of calcium signalling during infection with *S. maltophilia* in MMECs (Fig. 8G). Furthermore, the concentrations of the proinflammatory cytokines TNF-α and IL-1β were also reduced after inhibition by the calcium chelator (Fig. 7H and I). These results suggested that the calcium signalling pathway is a key target for *S. maltophilia* to exert its pathogenic effects.

### Inhibition of autophagy alleviates the inflammatory response induced by *S. maltophilia*

To validate the role of autophagy in the induction of mastitis and blood-milk barrier disruption by *S. maltophilia*, we treated MMECs with the autophagy inhibitor 3-MA and then infected them with *S. maltophilia*. The results showed that 3-MA increased the expression of p62 and inhibited the expression of LC3 II (Fig. 8A). In addition, inhibition of autophagy reversed the increase in the production of the proinflammatory cytokines TNF-α and IL-1β and the disruption of the blood-milk barrier induced by *S. maltophilia* (Fig. 8B–D). These results suggest that autophagy plays an important role in mastitis and blood-milk barrier function.

## Discussion

Mastitis is a common disease that affects both dairy herds and humans, resulting in excessive global economic losses in the dairy industry. A clear understanding of the pathogenesis of mastitis is helpful for effective disease prevention and treatment. It is thought that mastitis is induced by the invasion of pathogenic bacteria into the mammary gland through the mammary ducts. However, recent studies indicated that an endogenous gut-mammary pathway in dairy cows might also play an important role in mastitis. Our previous studies found that long-term feeding with a high-concentrate diet induced mastitis, and the abundance of *Stenotrophomonas* was significantly increased in both the rumen and milk. Furthermore, mastitis was induced by oral administration of *Stenotrophomonas* in lactating mice [4]. However, the mechanism by which *Stenotrophomonas* induces mastitis remains unclear.

Studies on the gut-brain axis, the gut-lung axis, the gut-liver axis and the gut-kidney axis revealed that gut microbiota disorders can cause harmful bacteria and metabolites present in the gut to migrate to the brain, lung, liver, kidney and other distal extraintestinal organs in an endogenous manner, causing related diseases [21–24]. In addition, given that substances in blood and lymph accumulate in the mammary gland during lactation, we hypothesize that enterogenic *Stenotrophomonas* may mediate the gut-mammary gland axis by migrating from the gut to the mammary gland and inducing mastitis. Under normal circumstances, GFP, as a neutral protein, does not affect the physiological characteristics of bacteria [25]. Thus, *S. maltophilia* was labelled with sf-GFP to test whether the bacteria could migrate from the gut to the mammary gland after oral administration. The results showed that oral administration of sf-GFP-*S. maltophilia* induced the inflammatory response of mastitis in mice and disrupted the gut barrier and the blood-milk barrier. It is well known that gut leakage is the pathological basis for the migration of bacteria and harmful substances from the intestine to the tissues and organs outside the intestine [26]. Evidence has shown that consumption of a high-fat diet (HFD) for only 1 week can drive bacterial translocation from the gut to the liver by destroying the gut barrier [27]. In addition, a study also suggested that gut leakage can promote the translocation of gut molecules derived from bacteria and enhance susceptibility to stress-induced apoptosis [28]. Similarly, disruption of the integrity of the blood-milk barrier is also an important mechanism by which exogenous substances enter the mammary gland. Therefore, we hypothesized that *S. maltophilia* may migrate from the intestine to the mammary gland via the damaged gut and blood-milk barriers, inducing mastitis. Here, sf-GFP-*S. maltophilia* was detected in various tissues and organs, including mammary gland tissues, mammary lymph nodes, mesenteric lymph nodes, lung, and serum. Although we did not trace the migratory path of *S. maltophilia* by in vivo fluorescence imaging, we demonstrated that enterogenic *S. maltophilia* can migrate from the gut to the mammary gland via an endogenous pathway.

To identify secreted signals underlying mastitis induced by *S. maltophilia*, we performed transcriptomic analysis of mammary gland tissues in mice orally administered *S. maltophilia*. The results showed that treatment with *S. maltophilia* significantly increased the expression of proteins related to calcium signals and the AMPK signalling pathway while reducing the expression of proteins related to tight junction protein cellular processes. In addition, intracellular Ca^2+^ levels also increased significantly under the action of *S. maltophilia*. Studies have shown that Ca^2+^ modulates both ROS generation and ROS clearance processes and thereby shifts the redox state [29], and ROS play an important role in the activation of autophagy by regulating the AMPK/mTOR signalling pathway [30]. In addition, recent studies have also indicated that Ca^2+^/AMPK/mTOR play important roles in the activation of autophagy [20, 31–33]. Autophagy is a process in which lysosomes degrade their cytoplasmic proteins and damaged organelles. Previous studies have shown that autophagy can lead to damage to tight junction proteins in the brain microvascular endothelium [34]. Others also indicated that autophagy induces the loss of tight junction proteins in the mammary gland through the accumulation of autophagosomes or the induction of cell apoptosis [35, 36]. Moreover, autophagy also plays a role in the development of mastitis. Evidence suggests that pathogens, such as *Streptococcus agalactiae*, induce autophagy through the PI3K/ATK/mTOR pathway in bovine mammary epithelial cells (BMECs) [37], and *Streptococcus lutetiensis* induces autophagy through oxidative stress in BMECs [38]. Additionally, taurine inhibits the inflammatory response induced by *Streptococcus uberis* by regulating autophagy in MAC-T cells [39]. In the present study, we found that oral administration of *S. maltophilia* promoted Ca^2+^/ROS/AMPK/mTOR/autophagy pathway activation; however, inhibition of calcium ions by EGTA reversed the changes in Ca^2+^/ROS/AMPK/mTOR/autophagy and increased the expression of tight junction proteins in the blood-milk barrier that were altered by *S. maltophilia*. In addition, inhibition of autophagy by 3-MA also reversed the changes in the inflammatory response and blood-milk barrier in MMECs given *S. maltophilia*. These results indicated that *S. maltophilia*-induced mastitis and the increase in blood-milk barrier permeability were associated with Ca^2+^/ROS/AMPK/mTOR/autophagy pathway activation.

## Conclusion

Our study indicates that mastitis not only results from infection by exogenous pathogenic microorganisms but also involves an enterogenic pathway that facilitates intestinal bacterial migration to the mammary gland. Our results demonstrate that enterogenic *S. maltophilia* migrates from the gut to the mammary gland to mediate the gut-mammary axis and activates the calcium-ROS-AMPK-mTOR-autophagy pathway, causing mastitis. In addition, the results make an important and innovative contribution to knowledge on the pathogenesis of mammary diseases, such as mastitis, and have important significance for the development of strategies to treat and prevent diseases in other organs induced by gut microbiota.

### Supplementary Information

**Additional file 1: Table S1.** Primers used in this study. **Table S2.** Blood routine examination. **Table S3.** Blood biochemical examination. **Fig. S1.** Superfolder-GFP (sf-GFP) fluorescent labelling of *S*. *maltophilia*. **Fig. S2.** Additional data from transcriptome sequencing results.

## Data Availability

All data can be found in the manuscript or in the supplementary material. Transcriptomic data were deposited in the US National Center for Biotechnology Information (NCBI) sequence read archive under accession number PRJNA983979.

## Abbreviations

AMPK: Adenosine 5'-monophosphate -activated protein kinase
APS: Ammonium persulfate
DAPI: 4',6-diamidino-2-phenylindole
ELISA: Enzyme linked immunosorbent assay
FBS: Fetal bovine serum
GFP: Green fluorescent protein
H&E: Haematoxylin and eosin
LB: Luria–Bertani
MMECs: Mouse mammary epithelial cells
MPO: Myeloperoxidase
mTOR: Mammalian target of rapamycin
OD: Optical density
PBS: Phosphate-buffered saline
sf-GFP: Superfolder-GFP
SARA: Subacute rumen acidosis
*S. maltophilia*: *Stenotrophomonas maltophilia*
TBST: Tris-buffered saline with 5% tween-20
