# Supplementary material for: Enterogenic Stenotrophomonas maltophilia migrates to the mammary gland to induce mastitis by activating the calcium-ROS-AMPK-mTOR-autophagy pathway
Source: J Anim Sci Biotechnol. 2023 Dec 20;14:157. doi: 10.1186/s40104-023-00952-y (PMC10731779; doi:10.1186/s40104-023-00952-y)
Supplement: Supplementary file 1 — Additional file 1: Table S1. Primers used in this study. Table S2. Blood routine examination. Table S3. Blood biochemical examination. Fig. S1. Superfolder-GFP (sf-GFP) fluorescent labelling of S. maltophilia. Fig. S2. Additional data from transcriptome sequencing results. [file 40104_2023_952_MOESM1_ESM.docx]

**Table S1** Primers used in this study

| **Gene** | **Primer** | **Sequence (5′→3′)** | **Product length, bp** |
| --- | --- | --- | --- |
| sf-GFP | Sense | ATGAGCAAAGGAGAAGAACTTTTCA | 717 |
|  | Antisense | CTATTTGTAGAGCTCATCCATGCCA |  |

**Table S2** Blood routine examination (*n* = 6)

|  | **Control (x̅±SEM)** | ***S.ma* (x̅±SEM)** | ***P*-value** |
| --- | --- | --- | --- |
| RBC | 7.87±0.53 | 7.85±0.15 | 0.9718 |
| MCV | 48.33±1.08 | 48.30±0.89 | 0.9833 |
| RDW% | 27.52±1.64 | 31.88±1.09 | 0.0507 |
| RDWa | 35.63±0.60 | 40.88±1.641 | 0.0133 |
| HCT | 37.83±2.081 | 37.96±1.027 | 0.9553 |
| PLT | 1,005.00±115.48 | 1,247.66±99.48 | 0.1425 |
| MPV | 7.18±0.22 | 7.83±0.31 | 0.1221 |
| WBC | 2.26±0.24 | 5.03±0.20 | < 0.0001 |
| HGB | 12.55±0.63 | 12.98±0.31 | 0.5503 |
| MCH | 16.13±0.35 | 16.53±0.23 | 0.3578 |
| MCHC | 33.46±0.23 | 34.25±0.20 | 0.0288 |
| LYM | 1.35±0.17 | 2.66±0.22 | 0.0008 |
| GRAN | 0.70±0.11 | 1.88±0.26 | 0.0016 |
| MONO | 0.20±0.03 | 0.48±0.04 | <0.0001 |

**Table S3** Blood biochemical examination (*n* = 6)

|  | **Control (x̅±SEM)** | ***S.ma* (x̅±SEM)** | ***P*-value** |
| --- | --- | --- | --- |
| ALB | 25.80±0.82 | 25.70±0.60 | 0.9236 |
| TPO | 53.93±1.90 | 58.31±1.63 | 0.1104 |
| GLO | 28.15±1.24 | 32.58±1.42 | 0.0402 |
| A/G | 0.90±0.04 | 0.95±0.03 | 0.0474 |
| ALT | 59.00±4.02 | 107.00±7.07 | 0.0001 |
| Cr | 19.80±1.47 | 20.00±3.20 | 0.9519 |
| BUN | 12.62±0.88 | 19.37±0.10 | 0.0005 |
| B/C | 624.35±103.10 | 970.52±240.70 | 0.2191 |
| Glc | 6.638±0.51 | 5.55±0.78 | 0.2722 |


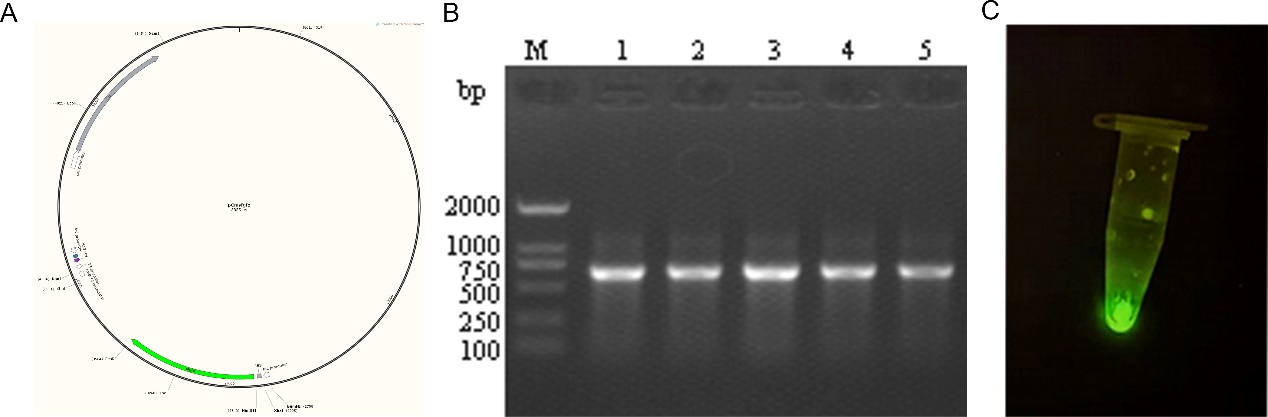


**Fig. S1** Superfolder-GFP (sf-GFP) fluorescent labelling of *S. maltophilia*. The plasmid containing sf GFP gene sequence was transferred into *S. maltophilia*. **A** Plasmids used to label *S. maltophilia*. **B** Agarose gel electrophoresis results of plasmid-transformed colonies. **C** The sf-GFP labeled maltophilus solution emits green fluorescence under 470 nm blue light

Fig. S1 A:


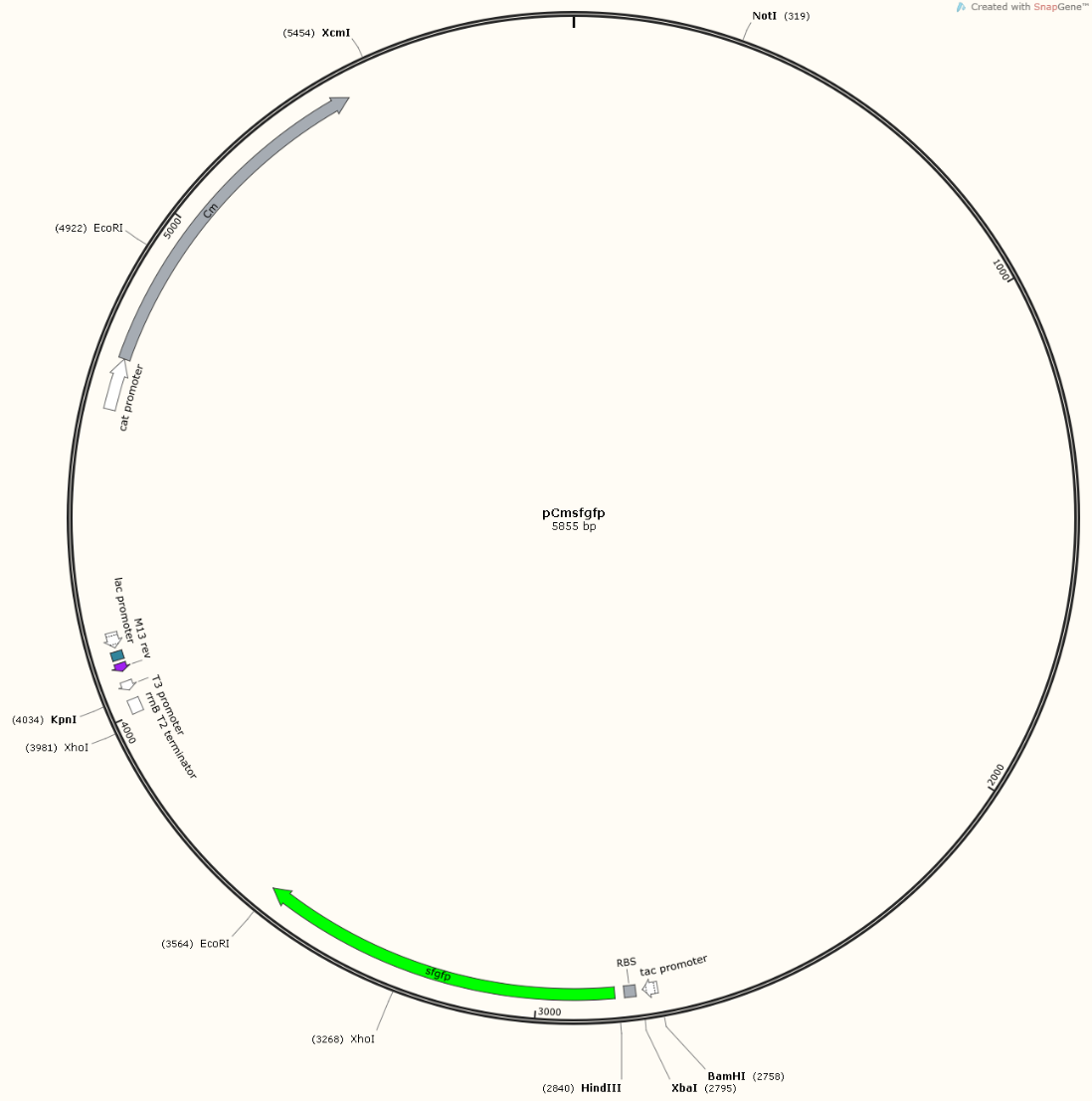


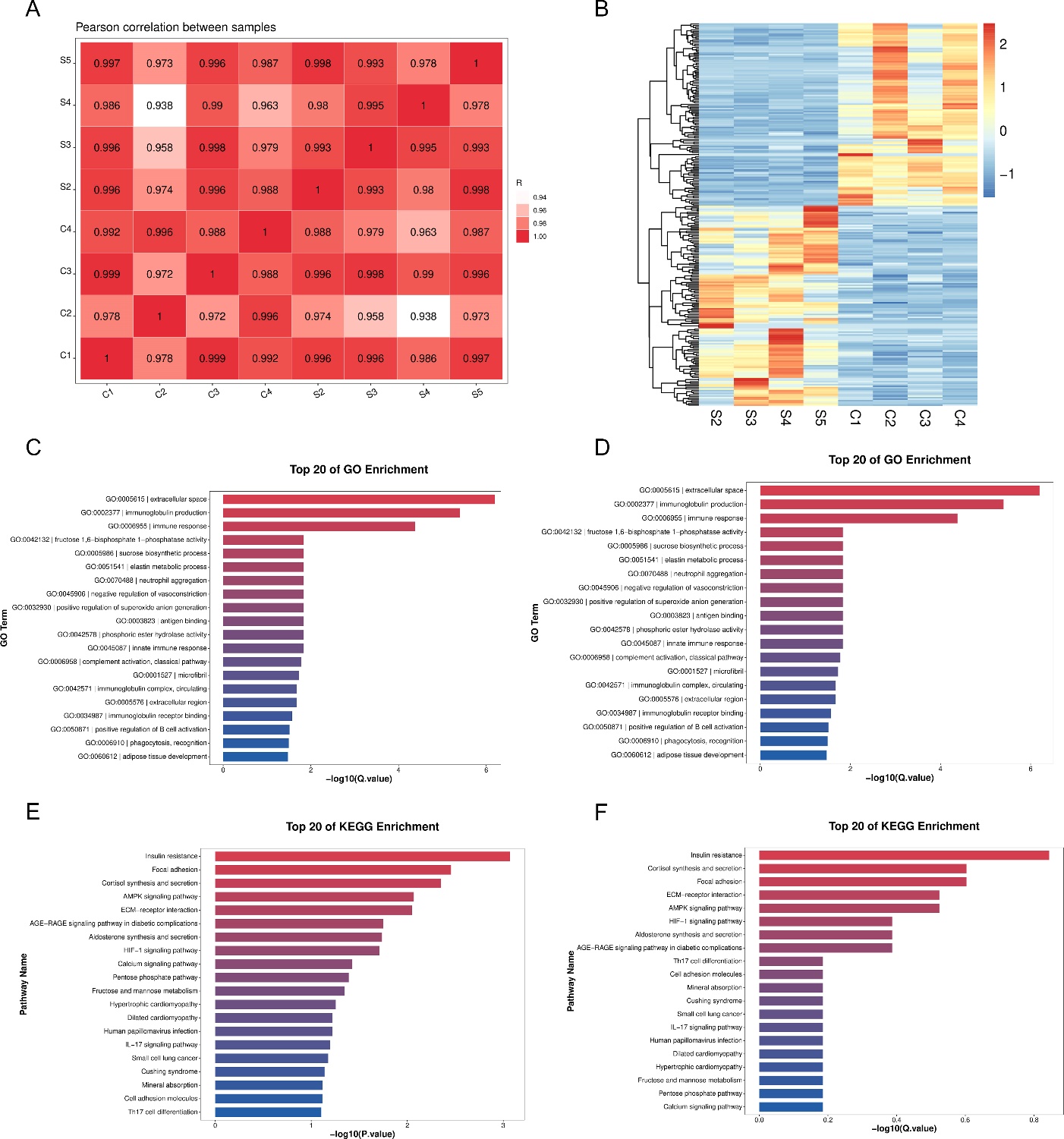


**Fig. S2** Additional data of transcriptome sequencing results. Mammary gland tissues were collected for transcriptome sequencing after the mice were treated with 10^10^ CFU *S. maltophilia* for a week. **A** Correlation analysis of samples. The inter organizational correlation ranged from 0.938 to 1. **B** Differential gene clustering heat map. According to the similarity of the gene expression profiles of the samples, the genes of the control group and the *S. maltophilia* treatment group were clustered. **C** GO enrichment bar graph of the first 20 GO entries with the lowest P value. **D** GO enrichment bar graph of the first 20 GO entries with the lowest *Q* value. **E** The first 20 KEGG pathways with the lowest *P* value were used to make KEGG enrichment bar graph. **F** The first 20 KEGG pathways with the lowest *Q* value were used to make KEGG enrichment bar graph
